# Deciphering the Molecular Nature of Ovarian Cancer Biomarker CA125

**DOI:** 10.3390/ijms130810568

**Published:** 2012-08-22

**Authors:** Florian Weiland, Karina Martin, Martin K. Oehler, Peter Hoffmann

**Affiliations:** 1 Adelaide Proteomics Centre, School of Molecular and Biomedical Science, University of Adelaide, Adelaide, SA 5005, Australia; E-Mails: florian.weiland@adelaide.edu.au (F.W.); karina.martin@adelaide.edu.au (K.M.); 2 Research Centre for Reproductive Health, Robinson Institute, University of Adelaide, Adelaide, SA 5005, Australia; E-Mail: martin.oehler@adelaide.edu.au

**Keywords:** CA125, MUC16, ovarian cancer, biomarker, mass spectrometry

## Abstract

The ovarian cancer biomarker CA125 has been extensively investigated over the last 30 years. The knowledge about the exact molecular nature of this protein, however, remains fragmented. This review provides an overview of the structural research regarding CA125, and presents an orthogonal verification method to confirm the identity of this molecule. The need for independent identification of CA125 is exemplified by several reports where mutually exclusive data concerning the existence of isoforms and the glycan moieties is presented. Mass spectrometry can overcome the pitfalls of a single detection/identification method such as antibody probing. Independent verification of CA125 identity in characterization studies will help establish a refined model of its molecular structure that will promote the development of new approaches for diagnosis, prognosis and therapy of ovarian cancer.

## 1. Introduction

The discovery of CA125 represented a milestone for ovarian cancer detection. In 1981, Bast *et al.* identified a murine monoclonal antibody, OC125, which reacted almost exclusively with ovarian cancer cell lines and cryo-preserved tissue of ovarian cancer patients [1]. An immuno assay for the detection of the OC125 antigen (CA125) in serum was soon developed and demonstrated a significant correlation between CA125 expression levels and the regression, stability or progression of epithelial ovarian carcinomas [2]. However, elevated serum concentrations were also found in 29% of patients with non-gynecological cancers as well as individuals presenting with benign conditions such as endometriosis, menstruation and pregnancy [2,3]. Due to the lack of specificity of CA125 a risk of malignancy index (RMI) is commonly used in clinical practice which combines CA125 levels, ultrasonographic findings and menopausal status of the patient. Using this approach 85% sensitivity and 97% specificity can be achieved when distinguishing benign from malignant ovarian disease [4]. However, this is not a suitable screening strategy for ovarian cancer as this disease has a low prevalence and requires a specificity of more than 99.6% to achieve an acceptable positive predictive value of 10% [5]. A large randomized controlled trial (RCT) of ovarian cancer screening (OCS) which is ongoing in the United Kingdom, involving 200,000 postmenopausal women (UK Collaborative Trial of Ovarian Cancer Screening, UKCTOCS, www.ukctocs.org) in a multimodality approach, estimates a woman’s risk of ovarian cancer (ROC) [6]. Here, an estimate on the basis of age and modified by the relative fit of the serial CA125 profile to the change-point model estimated from known cases is compared with the flat profile model estimated from known controls. Women who are found to have a high ROC then undergo screening by transvaginal ultrasonography. Preliminary results are promising but it remains unknown if this multimodality screening with CA125 will improve disease specific mortality.

In order to improve detection, considerable research has been directed at furthering the understanding of the functional and biochemical nature of CA125. Three decades of research unveiled the partial nucleotide and amino acid sequence, cellular localization and secretion, oligosaccharide structures and possible biological roles of CA125 during cancer and normal conditions. Unfortunately, the presented data is incomplete and contradictory. In many cases it is difficult to draw conclusions from the reports.

This article summarizes the currently available published data and conflicting views on the molecular nature of CA125. As this ambiguity may arise from antibody cross-reactivity, an orthogonal method of protein identification is required. Several studies employ mass spectrometry (MS) to independently verify antibody based CA125 detection. However, currently published mass spectrometric data does not satisfy established protocols and could therefore be unreliable. This highlights the need for high quality mass spectrometric data to enable reliable CA125 identification. Ultimately, the revised knowledge about the nature of CA125 may lead to the development of quantification techniques that will increase current sensitivities and specificities of ovarian cancer diagnosis, prognosis and progression.

## 2. Biological Function

The biological role for CA125 is still under investigation though several studies have demonstrated a relationship between CA125 and the immune system. In 2003 Kui Wong *et al.* proposed a role in immunity based upon the characterization of the *N*-oligosaccharide structures identified on CA125 [7]. This hypothesis was supported by the study of Patankar *et al.* where natural killer (NK) cell function was found to be inhibited in the presence of CA125 purified from OVCAR-3 cell culture [8]. Cell membrane-bound CA125 was subsequently shown to bind directly to NK cells derived from peripheral blood of ovarian cancer patients and pregnant women [9]. A potential link between the suppression of NK cells in feto-maternal tolerance and the immune evasion of ovarian cancers was therefore proposed [9]. Further investigation into the mechanism of interaction revealed that binding was established through the sialic acid-binding Ig-like lectin-9 (Siglec-9), an inhibitory receptor expressed on NK cells [10,11].

Furthermore, the *C*-terminus of CA125 has been shown to interact specifically with galectin-1 which, on its own, has been shown to be upregulated in various cancer-derived cell lines [12] (reviewed in [13]). Studies have also reported CA125 binding to mesothelin, a glycosylphosphatidylinositol-linked cell surface protein [14]. It is hypothesized that this interaction may play a role in detachment, attachment and localized invasion that is unique to ovarian cancer metastasis [15]. In addition, CA125 was described as a calcium-dependent protease, as the addition of EDTA to FPLC-purified CA125 prevented auto-proteolysis [16]. However, the presence of other proteins (e.g., proteases) in the high molecular weight CA125 FPLC fraction was not investigated. To date, knowledge about many functional aspects of CA125 remains incomplete. Further investigations are therefore required to elucidate the role(s) of this molecule in pathological and physiological conditions.

## 3. Nucleotide and Amino Acid Sequence

**Figure 1:**
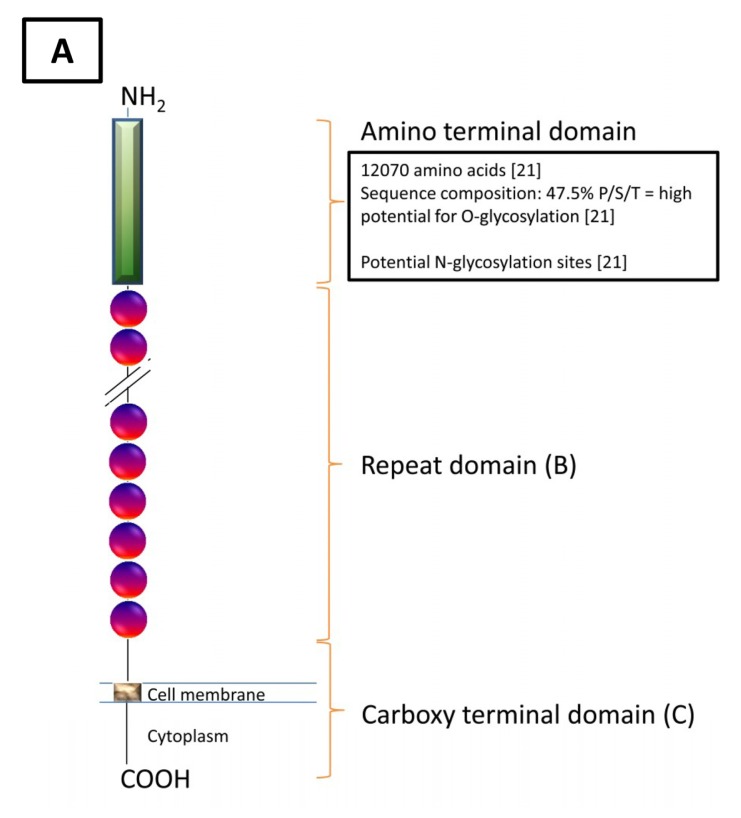

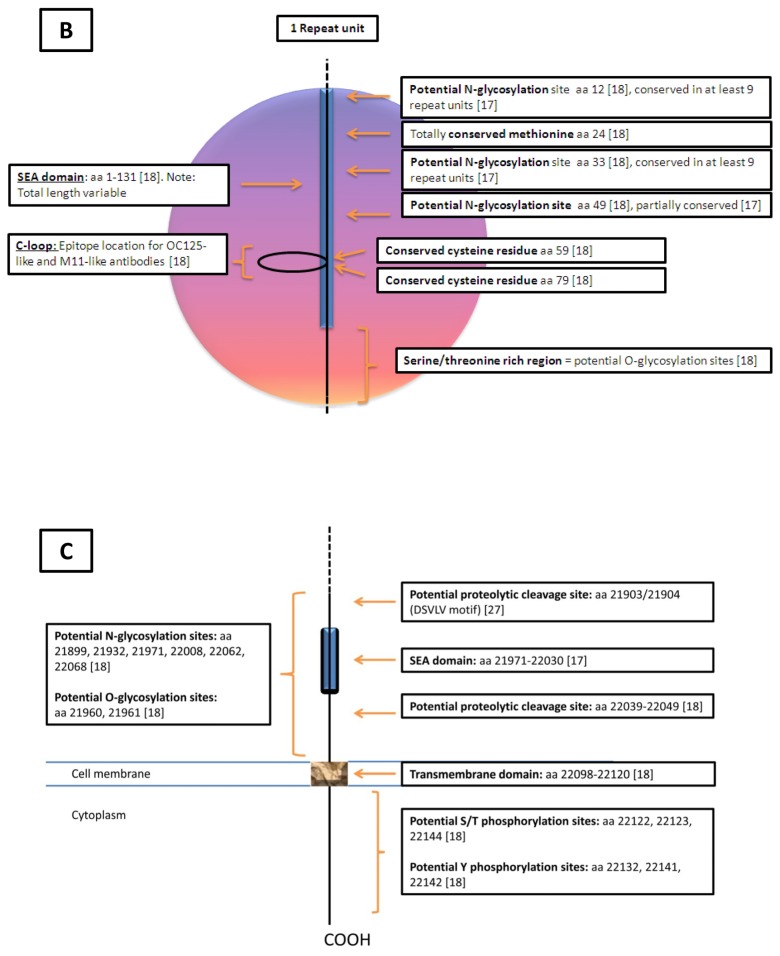
Schematic of CA125. (**A**) A diagrammatic overview of the molecular structure of CA125 as described by O’Brien *et al.* [18,21]; (**B**) A typical repeat unit of 156 amino acids and the possible features assigned to this sequence. At least 60 repeats have been identified, of which 45 are unique sequences, giving rise to a minimum domain length of 9800 aa [18]. Serine/Threonine rich region downstream of SEA domain was unspecified [18], *O*-glycosylation potential of a randomly chosen repeat unit (aa 14,255–14,410) was determined by submission to NetOGlyc 3.1 Server [25] (aa 131–156); (**C**) Carboxy terminal domain: aa 21,869–22,152 (length: 284 aa) [18] (aa numbering according to UniProt [26]).

The currently available genomic information on CA125 is based on two autonomous studies conducted in 2001 [17,18]. By screening a cDNA library generated using OVCAR-3 mRNA, Yin *et al.* identified a clone with a 5797 base pair (bp) nucleotide sequence [17] (reviewed in [19,20]). Although this region translated to a partial CA125 protein, it confirmed and revealed the existence of some key features. Importantly, they established the presence of partially conserved tandem repeats and were the first to describe the homology to the “sea urchin sperm protein, enterokinase, agrin” (SEA) domain. Yin *et al.* also postulated the existence of a transmembrane domain based upon a cluster of hydrophobic residues and a phosphorylation site based on a tyrosine phosphorylation consensus motif [17]. Furthermore, *O*-glycosylation was inferred due to the serine, threonine and proline content as well as *N*-glycosylation due to the asparagine content of the protein. These findings were supported and extended on by O’Brien and co-workers [18,21]. Here, a large transcript encoding CA125 was sequenced based upon a cyanogen bromide cleavage fragment of CA125 and a translated expressed sequence tag [16]. The resultant protein was described in three parts; an amino terminal domain, a repeat domain and a carboxy terminal domain (Table 1, Figure 1). The carboxy terminal domain reported by O’Brien *et al.* was homologous to the findings of Yin *et al.* [17,18,20]. One feature described only by O’Brien was the existence of a potential proteolytic cleavage site at amino acid residues 171–181 [18].

As described previously, the repeat domain consists of partially conserved tandem repeats that are 156 amino acids in length. The SEA domain was noted to constitute the first 131 amino acids of this region [18]. However, the amino acid sequence exhibiting a SEA domain is variable in length as the main feature of the consensus sequence is a “strand-strand-helix-strand-strand-helix” secondary structure motif and is unaffected by small sequence gaps between these units [24]. Therefore, as most CA125 tandem repeats are polymorphic, the SEA domain may not necessarily be 131 amino acids long. This is exemplified by the identification of a SEA domain consensus sequence with a length of 59 amino acids located in the non-tandem repeat region of the *C*-terminal domain [17]. Furthermore, the repeat domain contains a highly conserved methionine at position 24 as well as two highly conserved cysteine residues at position 59 and 79 [18]. These cysteine residues in the repeat domain were hypothesized by O’Brien to form a 19 amino acid loop to which the M11 antibody binds. Additionally, O’Brien demonstrated a loss of binding of the M11 antibody to a recombinant repeat domain when digested with Asp-N or Lys-C as this would have disrupted the cysteine loop [18].

In the study by O’Brien *et al.*, 45 unique and 15 redundant tandem repeat sequences were identified [18]. It was concluded that CA125 consists of at least 60 tandem repeats. However, due to difficulties associated with sequencing repeat regions some may be wrongly positioned or unknown, leaving the exact number of repeats undefined [18]. Furthermore, O’Brien states that the genome database is deficient in repeat information as repetitive sequences were potentially deleted during the compilation process of the human genome [18]. This suggests that the currently available nucleotide sequence of CA125 may not be correct.

The amino terminus was first described as a 1637 amino acid domain coded by 5 exons [17,18]. This was later revised by O’Brien *et al.* where an additional 37,700 nucleotides were sequenced from chromosome 19 [21]. Acquisition of the complete amino domain was confirmed by the identification of a Kozak translational initiation sequence as well as supporting Northern blot and mRNA expression data [21]. The main feature of this domain is the abundance of serine and threonine residues, which represent many potential sites of *O*-glycosylation, and disperse asparagine residues as potential sites of *N*-glycosylation [21].

The elucidation of the almost complete CA125 nucleotide sequence represented a significant leap in understanding. It enabled clarification of known and/or suspected features such as glycosylation (amino domain), multivalent antibody binding (repeat domain) as well as anchorage to and release from the membrane (carboxy domain) [12,27]. The findings also raise questions regarding the generation of lower molecular weight isoforms. Only the existence of a CA125 precursor protein of approximately 400 kDa has been found to exist in the cytoplasm of OVCAR-3 cells prior to protein maturation [28]. CA125 variants are also thought to arise from proteolytic cleavage. Proteins such as MUC1 have a proteolytic cleavage motif, GSVVV, in their SEA domain [29,30], however the presence of a motif with a similar consensus sequence has only been confirmed in one SEA domain of CA125 thus far [22]. A cluster analysis of a human CA125 sequence, containing the first 12 tandem repeats and a murine homolog to the *C*-terminus of CA125, was performed by Maeda *et al.* [22]. Interestingly, a DSVLV sequence in the second SEA domain from the cytosolic side of CA125 was clustered with the GSVVV sequence. Therefore, it was concluded that proteolytic cleavage might also occur in this domain of CA125 [22]. However, the formation of further proteolytic fragments of CA125, by the mechanism of the DSVLV or GSVVV sequence, in additional SEA domains has not been reported. Furthermore, O’Brien *et al.* suggested that the formation of alternative splicing products of this protein seems unlikely, based on the inherent variation of the five exons encoding for each repeat sequence [18]. Therefore, a discrepancy exists between antibody findings of low molecular weight isoforms and the protein sequence data (discussed below).

## 4. Antigenic Determinants

Numerous antibodies have been generated against CA125 since the initial discovery in 1981. These antibodies are classified as OC125-like (group A), M11-like (group B) or OV197 (group C) depending on the antigenic determinant recognized [31]. These antibodies are further subdivided into groups A1–A4 (OC125-like), B1/B2 (M11-like) and C1/C2 (OV197-like) [32,33]. It is important to note that although there are two antibodies classified as OV197-like, the antigenic recognition behavior of OV197 is different to that of 7C12 [32]. Extensive investigation into the epitope recognition of the different class antibodies to CA125 has been performed by the International Society of Oncology and Biomarkers (ISOBM) TD-1 workshop [31]. Cross-inhibition and immunometric assays have been used to examine the various epitopes on CA125 isolated from normal abdominal fluids, cervical mucus, cell culture and ascites [32]. From this the importance of antibody class combinations for accurate detection of CA125 was revealed. Furthermore, differential antibody interaction with CA125 isolated in low-molecular-weight fractions was also highlighted. Here, different immunometric assay combinations resulted in different CA125 activity levels in those fractions [32]. It was noted that the difference in assay results could be due to the different behavior of the antibody combinations towards the low-molecular-weight fractions of CA125 preparations [32]. Conversely, fractions containing high-molecular-weight CA125 yielded similar CA125 activity levels regardless of the antibody pair used in the immune-assay. As high-molecular-weight forms of CA125 are the major component in most samples this phenomenon should, therefore, not interfere with the immunometric assays even if the standards used do not have the same composition as the sample [32].

OC125-like and M11-like antibodies are proposed to detect a cysteine enclosed loop (C-loop) structure in the repeat domain [18,31,32] with a paucity of glyco-structures in the immediate proximity [18]. Extrapolated from the C-loop of a murine derived CA125 repeat domain homolog, Maeda *et al.* suggested that this antigenic region forms a β-sheet structure [22]. This was later confirmed with a synthesized 21 amino acid long human CA125 antigenic domain [23]. Here, a serine in position 8 of the loop was found to be essential for structural formation [23]. However, it is important to note that this serine at position 8 is conserved in the repeat sequences, throughout human CA125, in only around 25% of the cases. In approximately 60% of repeat sequences a proline is found at position 8, which results in random coil formation [23].

The fourth report from ISOBM TD-1 workshop described the use of a recombinant sequence of the 11th repeat from the *N*-terminus (R11) to test the binding capacity of various OC125-like, M11-like and OV197-like antibodies [33]. It was shown that the binding of the various monoclonal antibodies differed between the groups and subgroups. Within group A, subgroups A3 and A4 showed high binding to R11. Group B also displayed high reactivity with the exception of four investigated M11-like antibodies that showed low to no binding [33]. Additionally, group C antibodies had shown only little or no reactivity [33]. From those results it was concluded that the poor or absent binding of some antibodies towards R11 is unlikely to be due to a disparate sequence of this particular repeat to the other +60 repeat sequences, as there are only three main epitope families identified to date [33]. This observation was thought to be, more likely, a result of the single R11 repeat not exhibiting an optimal conformation as it is no longer in its native context [33]. This was exemplified by the binding behavior of OC125, which binds poorly to R11 [33] yet shows strong binding to a recombinant 3 tandem repeat form of CA125 [34].

Furthermore, glycosylation of CA125 has also been shown to be important for high affinity antibody binding. Deglycosylation with PNGase F led to lower affinity binding of the OC125 antibody to CA125 [7]. However, the exact influence glycosylation has on the binding affinity of the different antibody classes to CA125 is unknown.

## 5. Forms and Variants

Several different masses corresponding to CA125 have been reported in the literature. A protein with a mass over 1000 kDa as well as proteins with a lower molecular mass ranging from 200–400 kDa, 130 kDa, 205 kDa and 55 kDa have been observed depending on the source material analyzed [35–37] (reviewed in [38]). A number of these studies have also reported a modular structure of CA125, which can be broken down into 200 kDa, 70 kDa and 50 kDa species [35,37]. These forms of CA125 have been referred to as splice variants, which give rise to amino acid sequences with a molecular mass between 49 and 1500 kDa [22,39].

However, studies by Lloyd and Yin found no evidence for the low molecular weight products reported by other groups using an OVCAR-3 cell line [28,40]. Later they reported a precursor form of CA125 of approximately 400 kDa in an OVCAR-3 cell line, from which the mature form of the protein becomes assembled [28]. It is noted that only the mature protein form is released into the surrounding medium [28]. Additionally, the low molecular weight forms reported by other groups were assessed as possible degradation products by using trypsin [28]. This resulted in no defined, prominent molecular weight bands on a SDS-PAGE gel [28]. Furthermore, our research has not found evidence of low molecular weight forms of CA125 in human ascites [41]. Here, cross-reactivity of the employed M11-like and OC125-like antibodies was shown by two-dimensional western-blotting, mass spectrometric identification and spiking experiments. Therefore, the existence of lower molecular weight forms of CA125 remains uncertain.

## 6. Oligosaccharide Moieties

Mucins such as CA125 are large proteins associated with extensive glycosylation that is important for structure and function (reviewed in [42]). In addition to the contradicting views regarding the molecular weight forms of CA125, the types of glycosylation reported are also controversial. Davis *et al.* identified CA125 as protein with, in comparison to other mucins, low carbohydrate content of 24%, constituting mostly mannose and N-linked glycans [35], while Hanisch *et al.* described CA125 as consisting of 68% carbohydrates, essentially mannose free and O-linked glycans [43].

A third view was presented by Nagata *et al.* in 1991 [44]. They found CA125 to be a glycosylphosphatidylinositol (GPI) anchored molecule with N- and O-linked glycan structures [44]. Independently, a study by Kui Wong *et al.* also found N- and O-linked glycans attached to CA125 [7]. Interestingly, one identified structure was identical to an unusual glycostructure expressed by uromodulin [7,45]. In light of our recent results these contradicting reports may arise from cross-reactivity of the M11-like and OC125-like antibodies with other proteins [41]. This may have lead to the characterization of proteins that were wrongly assigned as CA125.

Additionally, aberrant glycosylation of CA125 has been reported for various diseases. The study of Pastan *et al.* demonstrated increased presentation of Lewis y blood group tetrasaccharide (Le^y^) on ovarian cancer tissue compared with normal ovarian tissue [46]. This Le^y^ modification, expressed in 75% of ovarian cancer tissue specimens, was identified as a part of the CA125 glycan structure [47]. Microheterogeneities in CA125 glycosylation may represent a possible diagnostic indicator, according to Jankovic *et al.*, as differential glycosylation of CA125 derived from amniotic fluid and OVCAR-3 cells was demonstrated [48]. Furthermore, Mitic *et al.* reported differential binding of CA125 to a set of sialic acid-binding Ig-like lectins (Siglecs) depending on the source of the protein. A preference for Siglec-9 binding was evident for CA125 derived from an OVCAR-3 cell line [49]. Interestingly, this interaction has been shown to be necessary for the binding to and subsequent suppression of NK-cell function [8–11].

## 7. Mass Spectrometric Identification of CA125

Isolation of CA125 for the analysis of its molecular features represents a challenge for researchers, as routine high resolution methods such as SDS-PAGE are not ideal for very high molecular weight analytes. Typically, size exclusion chromatography (SEC) is used for CA125 purification; however the separation efficiency is limited and results in cross-contaminations [16]. Additional methods used for the prefractionation of CA125 include immuno-precipitation, affinity chromatography or a combination of these methods that are associated with similar disadvantages [12,16,28,48,50]. Subsequent identification of CA125 in the derived fractions is therefore necessary for reliable downstream analysis. However, CA125 identification solely based on antibody probing alone can lead to false-positive protein assignment as a result of antibody cross-reactivity [41]. Thus, characterization of these secondary proteins may lead to erroneous assignment of molecular attributes to CA125. Several studies show that mass spectrometry (MS) offers a suitable solution for independent CA125 identification (discussed below). It is, however, notable that the published protein sequence for CA125 is incomplete [17,18,21]. Although this may result in some peptide identifications being missed by search algorithms, mass spectrometric identification of CA125 is a viable approach that can complement antibody based identification methods.

The first peptide mass fingerprinting data on CA125 was described in 2003 where 16 masses, corresponding to peptides of a 1148 amino acid form of CA125 from HeLa cells, were detected [12]. This enabled the identification of CA125 as a binding protein of galectin-1. In a later study, Jankovic *et al.* identified an approximately 200 kDa form of CA125 in first trimester human placental extract based on 36 experimentally detected masses [51]. Similarly, Milutinovic *et al.* reported identification of 46 masses that matched to CA125 in human amniotic fluid [52].

Tandem mass spectrometric data on CA125 was first reported by Kui Wong *et al.*, confirming the identity of CA125 in a purified sample prior to oligosaccharide characterization [7]. Although only a single peptide was reported, this identification was supported by peptide mass fingerprinting where 11 experimentally derived masses were matched to theoretical peptides. Analyses of the tear proteome by tandem MS lead to the identification of 491 proteins including CA125 [53]. This was the first mass spectrometric data of CA125 that included statistical analysis and associated metadata for eight peptides of this protein. However, the molecular weight range of the fraction in which CA125 was identified was not stated. Using Fourier-transform ion cyclotron resonance (FT-ICR) tandem mass spectrometry Andersch-Bjorkman *et al.* detected 31 peptides from a 2–3 MDa form of CA125 derived from mucus of the human cervical opening [54]. Davies *et al.* also reported the identification of a high molecular weight form of CA125 in human tracheal secretions [55]. Recently, we have also presented mass spectrometric data including statistical analysis and metadata of 21 peptides of a high molecular weight species of CA125 [41]. These data were used to confirm the identity of CA125 in the ascites of ovarian cancer patients as a high molecular weight protein.

To date, mass spectrometric data of CA125 is still quite rare and seldom reported in a way that satisfies the “Minimum Information about a Proteomics Experiment” (MIAPE) standards [56–58], even after 2007. MIAPE standards have been implemented to avoid confusion about mass spectrometric experiments as well as enhance reproducibility and reliability of MS data. It is evident that the quality of mass spectrometric data regarding CA125 varies greatly and the majority of studies do not allow for independent assessment. Furthermore, statistical analysis of the MS-generated data is seldom presented. This is a major issue, especially for CA125, as the potential for incorrect peptide matches rises with increasing length of protein sequence [59].

Additionally, matching experimentally found masses to theoretical masses without the usage of statistical tools is prone to produce false-positive identifications. Schober *et al.* demonstrated that when a mass accuracy window greater than ± 10 ppm is used, numerous peptides from different proteins with similar masses can be assigned to the incorrect protein [60]. This means that CA125 identification without supporting statistical analysis is unreliable and potentially false-positive.

## 8. Conclusion

CA125 is heavily relied upon for diagnosis and prognosis of epithelial ovarian cancer; however the molecular characteristics are poorly understood and are the subject of ongoing investigations. This review summarizes and evaluates the available data regarding the molecular features of CA125. While the nucleotide sequence of this protein was independently cloned by two groups and showed an almost complete homology between the overlapping parts, other molecular features like predominant type of glycosylation and isoforms are not only disputed but mutually exclusive. Although mass spectrometry has already been shown to be a valid option to clarify these ambiguous findings, most data generated by this method, and published so far, does not satisfy established reporting standards. This review highlights the importance of high quality mass spectrometric data to independently verify the identity of the investigated protein. This would ensure that CA125 identified by antibody-dependent methods are not cross-reactive proteins. Therefore, the ambiguous views on the glycan structure as well as the controversial low molecular weight forms can be clarified. This greater understanding may help improve established measurement techniques for assaying CA125 serum levels. Alternatively, novel detection systems that are independent of antibodies, such as selective reaction monitoring mass spectrometry (SRM), could also be applied for CA125 quantitation in patient samples. SRM has already been shown to be a feasible approach to quantify proteins in plasma [61]. Furthermore, detailed structural information on CA125 will enable the development of new and more refined molecularly targeted antibody therapies which already show great clinical potential and warrant further research [62–65].

## Figures and Tables

**Table 1:** Features described from the CA125 amino acid sequence based on the data of O’Brien [18,21], Yin [17], Maeda [22] and Berman [23]; Amino acid (aa) number (numbering derived from [18]) indicates the position of those residues as they appear within the respective domain. bp: base pairs.

Domain	Carboxy Domain	Repeat Domain	Amino Domain
Features			
Exons	9	5	9
Nucleotides (bp)	14,000	Single: 1900 Cumulative: Unknown	50,950
Amino Acids (aa)	284	Single: 156 Cumulative: >9360	12,068
Molecular Characteristics	Stop CodonPoly A Signal Potential Transmembrane Region (aa 230–252) Potential Cytoplasmic Region (aa 256–260) Potential Proteolytic Cleavage Sites (aa 171–181 [18] and aa 35/36 [22])	SEA domain Cysteine loop (aa 59,79) [23] Conserved Methionine (aa 24) Serine/Threonine/Proline Rich	Serine/Threonine Rich(Abundant *O*-glycosylation Potential, *N*-glycosylation Potential)
